# Aggressive angiomyxoma in the inguinal region: a case report

**DOI:** 10.1186/1752-1947-4-396

**Published:** 2010-12-08

**Authors:** Takeshi Kondo

**Affiliations:** 1 Division of Legal Medicine, Department of Community Medicine and Social Healthcare Science, Kobe University Graduate School of Medicine, 7-5-1 Kusunoki-cho, Chuo-ku, Kobe 650-0017, Japan

## Abstract

**Introduction:**

Aggressive angiomyxoma is a rare myxoid mesenchymal tumor of the pelvis and perineum, which occurs almost exclusively in adult women. The tumor is especially rare in men.

**Case presentation:**

We report the case of a 68-year-old Japanese man with a slowly growing inguinal swelling. At surgery, a huge mass in the soft tissue of the inguinal region was found, not involving the adjacent organs. The morphologic picture was compatible with aggressive angiomyxoma of the inguinal region.

**Conclusions:**

Aggressive angiomyxoma is a very rare, locally infiltrative neoplasm. Thus, after surgery, close follow-up is needed because of a high risk of local recurrence.

## Introduction

Aggressive angiomyxoma is a rare mesenchymal tumor of the pelvis and perineum that occurs almost exclusively in adult women [1]. It preferentially arises from the soft tissue of the pelvic region, perineum, and genital area. Its incidence is approximately sixfold higher in women, and 24 male cases have been reported in the literature [1]. The tumor is usually locally infiltrative and has a high rate of local recurrence after surgical excision [1]. The adjective "aggressive" emphasizes the neoplastic character of the blood vessels, its locally infiltrative nature, and the high risk of local recurrence, not indicating a malignant potential of the lesion. Rarely, this tumor appears in men, simulating inguinal hernia, testicular neoplasm, spermatic cord neoplasm, hydrocele, or spermatocele [2,3].

## Case presentation

**Figure 1:**
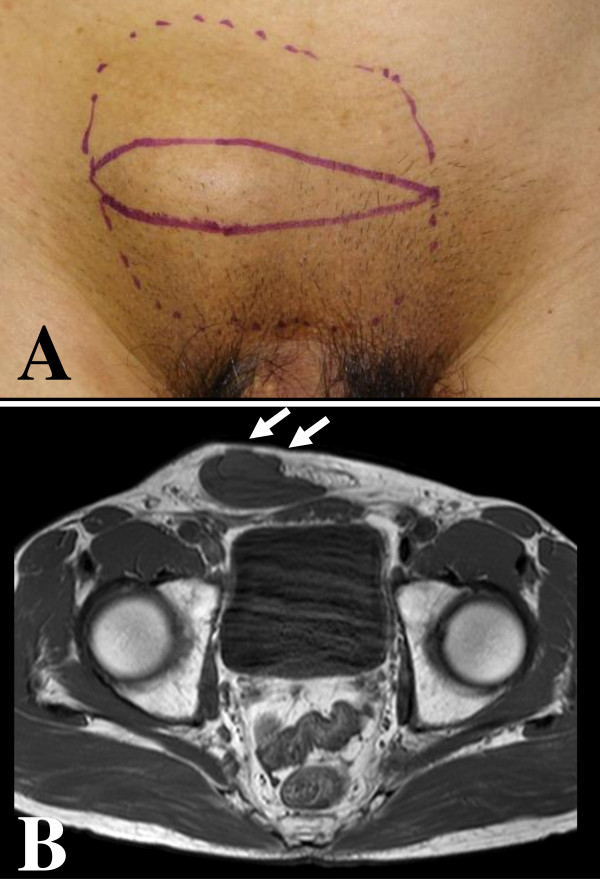
**A slowly growing swelling of the soft tissue in the inguinal region**. **(a) **Swelling of the soft tissue in the right inguinal region was observed. **(b) **Magnetic resonance imaging revealed that the lesion (arrows) was isointense relative to muscle on the T_1_-weighted image.

**Figure 2:**
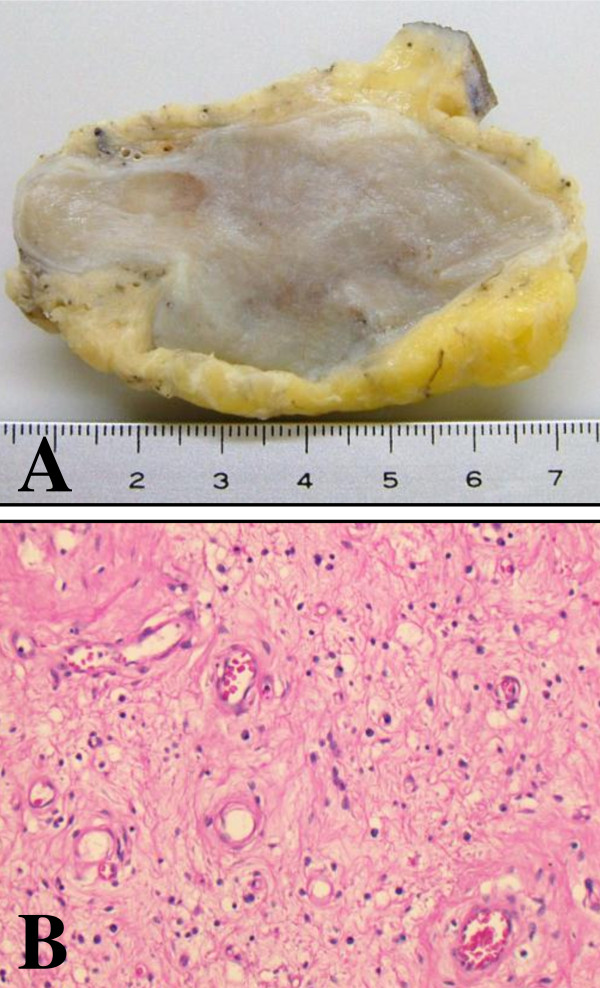
**Macroscopic and microscopic findings of the lesion**. **(a) **Macroscopic finding of the tumor. The tumor measured 7.5 cm and had a relatively clear margin. The cut surface of the tumor was smooth, homogeneous, and gray-white without necrosis or hemorrhage. **(b) **Microscopic findings of the lesion (hematoxylin and eosin stain, ×200). The tumor was composed of spindle cells and blood vessels with myxoid stroma.

A 68-year-old healthy Japanese man presented with a slowly growing swelling of the soft tissue in the inguinal region (Figure 1). The duration of symptoms was about five years. At surgery, a large encapsulated mass (7.5 cm) was found, not involving the adjacent structure. The tumor was easily removed, as it was discrete and without adhesions. The cut surface of the tumor was smooth, homogeneous, and gray-white (Figure 2a). Histologically, it was a paucicellular (hypocellular) tumor composed of fibrotic and myxoid areas, showing a sparse population of spindle-shaped tumor cells without significant cytologic atypia or mitosis (Figure 2b). Foci of thick-walled blood vessels of various sizes were identified. The tumor cells were positive for CD34, and negative for α-smooth muscle actin and desmin. The tumor cells were negative for hormone receptors (ER and PgR). Chronic inflammatory cells were found scattered in the stroma. The morphologic picture and the immunostain were compatible with aggressive angiomyxoma in the inguinal region. The operation itself was uneventful and, on follow-up, no signs of recurrence have appeared for about one year.

## Discussion

Since 1983, when aggressive angiomyxoma was first described by Steeper *et al*., about 100 cases in both sexes (including 24 men) were reported worldwide [1]. It often occurs in middle-aged patients (mean age, 46 years) [1]. Occurrence of aggressive angiomyxoma in men is extremely rare, and, in men, aggressive angiomyxoma is usually derived from the pelviperineal interstitial tissue involving the scrotum (38%), spermatic cord (33%), perineal region (13%), and intrapelvic organs (8%) [1].

Macroscopically, its typical cut-surface appearance is a large, grossly gelatinous, and locally infiltrative tumor. Microscopically, the stroma is rich in collagen fibers with a prominent vascular component, including many thick-walled vessels. The differential diagnosis includes angiomyoblastoma, myxoid neurofibroma, myxoma, spindle cell lipoma, and myxoid liposarcoma [1,2]. Immunohistochemically, the stromal cells of the tumor show strong positivity for vimentin and variable positivity for desmin, α-smooth muscle actin, and CD34 [2,4].

Immunohistochemical studies have revealed that tumor cells are immunoreactive for no specific marker. Male angiomyxoma may be positive for estrogen and progesterone receptor [2]. The tumor cells in this case, however, were negative for the two markers.

Cytogenic analysis reveals chromosomal translocation involving chromosomes 8 and 12, associated with rearrangement of the *HMGIC *gene [1].

Surgery is the principal first-line treatment to date and, because of the high risk of local recurrence, a long-term postoperative follow-up with either ultrasound (US) or computed tomography (CT) is recommended [1]. The recurrence may be attributed to incomplete tumor resection, because of the infiltrating nature, and the absence of a definite capsule. The earliest recurrence has been reported as appearing nine months after surgery [5]. No distant metastasis, however, has been reported.

## Conclusions

In conclusion, aggressive angiomyxoma is a very rare neoplasm that is more predominant in women. After surgery, close follow-up is needed because of the high risk of local recurrence.

## Competing interests

The authors declare that they have no competing interests.

## Authors' contributions

TK performed histologic examination, analyzed the case, and wrote the manuscript.

## Consent

Written informed consent was obtained from the patient for publication of this case report and accompanying images. A copy of the written consent is available for review by the Editor-in-Chief of this journal.
